# Obstructive Sleep Apnea and Cardiovascular Risk: The Role of Dyslipidemia, Inflammation, and Obesity

**DOI:** 10.3389/fphar.2022.898072

**Published:** 2022-06-15

**Authors:** Marija Zdravkovic, Viseslav Popadic, Slobodan Klasnja, Natasa Milic, Nina Rajovic, Anica Divac, Andrea Manojlovic, Novica Nikolic, Filip Lukic, Esma Rasiti, Katarina Mircetic, Djordje Marinkovic, Sofija Nikolic, Bogdan Crnokrak, Danica Popovic Lisulov, Sinisa Djurasevic, Maja Stojkovic, Zoran Todorovic, Ratko Lasica, Biljana Parapid, Predrag Djuran, Milica Brajkovic

**Affiliations:** ^1^ University Clinical Hospital Center Bezanijska Kosa, Belgrade, Serbia; ^2^ Faculty of Medicine, University of Belgrade, Belgrade, Serbia; ^3^ Institute for Medical Statistics and Informatics, Faculty of Medicine University of Belgrade, Belgrade, Serbia; ^4^ Department of Internal Medicine, Division of Nephrology and Hypertension, Mayo Clinic, Rochester, MI, United States; ^5^ Faculty of Biology, University of Belgrade, Belgrade, Serbia; ^6^ School of Medicine, University of Belgrade, Belgrade, Serbia; ^7^ Clinical Center of Serbia, Belgrade, Serbia

**Keywords:** obstructive sleep apnea, dyslipidemia, inflammation, echocardiography, cardiovascular risk

## Abstract

**Introduction:** The present study aimed to establish the role of lipid abnormalities and inflammatory markers for developing cardiovascular risk, as well as to address the importance of obesity as a common comorbidity in patients with obstructive sleep apnea (OSA).

**Methods:** The study was conducted as a prospective cohort study including 120 patients with newly diagnosed OSA between 2019 and 2020, at University Clinical Hospital Center “Bezanijska kosa”, Belgrade, Serbia. The diagnosis was established by polysomnography. In all patients, sociodemographic data, respiratory, lipid, and inflammatory parameters were collected and complete echocardiographic study and 24-h blood pressure monitoring were performed.

**Results:** The mean patient age was 55.7 ± 13.8 years. Study population was mostly male (70.0%) and obese (56.7%). At least 30 apneas or hypopneas per hour were present in 39.0% of patients. A strong positive correlation was found between OSA severity and BMI (r = 0.562, *p* < 0.001), both associated with lipid, inflammatory and respiratory parameters, and cardiovascular profile of patients with OSA (*p* < 0.05 for all). Echocardiographic study and 24-h blood pressure monitoring parameters were in turn correlated with lipid and inflammatory markers (*p* < 0.05 for all).

**Conclusion:** The results of this study support the important role of dyslipidemia and inflammation, as well as coexistence of obesity in the pathogenesis of numerous conditions linked with an increased risk of cardiovascular morbidity and mortality in patients with OSA.

## Introduction

Obstructive sleep apnea (OSA) is characterized by repetitive episodes of apnea due to upper airway obstruction occurring during sleeping hours. These episodes cause intermittent hypoxia which leads to oxidative stress and increased free radicals production (Eisele et al., 2015). This is important as it can induce various pathophysiological mechanisms responsible for endothelial dysfunction, increased sympathetic activity, vascular inflammation, metabolic syndrome, and platelet activation (Eckert and Malhotra, 2008). Metabolic syndrome and dyslipidemia are common disorders in patients with OSA. It is shown that one-third of all patients with OSA have metabolic syndrome with an almost identical distribution among men and women, especially in obese (Jean-Louis et al., 2008). Obese patients also tend to have severe forms of OSA, as it is shown that change in weight is directly proportionate to sleep disordered breathing (Jehan et al., 2017). Increased triglyceride levels and decreased HDL cholesterol are the most common findings in obese patients with obstructive sleep apnea (Karkinski et al., 2017). Additionally, individuals with metabolic syndrome have several concurrent disorders including diabetes mellitus and hypertension which are independent risk factors associated with cardiovascular morbidity and mortality (Floras, 2018). Recurrent hypoxia and the excitation of the sympathetic nervous system can also trigger an inflammatory response that may affect the whole vasculature and myocardium, leading to an increased susceptibility for accelerated atherosclerosis and its complications (Kheirandish-Gozal and Gozal, 2019). Induced oxidative stress can also change the composition of the extracellular heart tissue matrix, which can result in the increased prevalence of systolic and diastolic dysfunction and later development of heart failure (Sanderson et al., 2020). It is important to note that individuals with OSA do not only have an increased risk of developing cardiovascular comorbidities but also have worse outcomes related to cardiovascular disease (Javaheri et al., 2017). Obstructive sleep apnea increases the risk of heart failure by 140%, the risk of stroke by 60%, and the risk of coronary artery disease by 30% (Shahar et al., 2001). Atherogenesis in patients with OSA starts soon after the onset of sleep apnea, which is significant as these patients are often untimely diagnosed (Jean-Louis et al., 2009). This is why it is crucial to identify credible predictors of increased cardiovascular risk, set forehand diagnosis, and start a proper therapy to prevent future adverse cardiovascular events.

The present study aimed to establish the role of lipid abnormalities and inflammatory markers for developing cardiovascular risk, as well as to address the importance of obesity as a common comorbidity in patients with obstructive sleep apnea.

## Methods

The study was conducted as a prospective cohort study including 120 patients with newly diagnosed OSA during the period between May 2019, and May 2020, at the Department of Pulmonology, Center for diagnostics and treatment of obstructive sleep apnea, of the University Clinical Hospital Center “Bezanijska kosa”, Belgrade, Serbia. This study represents an additional prospective analysis of the previous study by Popadic et al. (2014). All participants over 18 years of age who had been referred for polysomnography (PSG) due to suspected sleep-related breathing disorders and with at least 3.5 h of sleep were included in the study. The exclusion criteria were the impossibility to perform polysomnography, unstable vital signs, major behavioral or neurological disorders, the use of medications that could affect sleep or autonomic nervous system function. All participants provided written informed consent.

### Polysomnography

To establish the diagnosis of OSA and its severity, the polysomnographic study was performed with the Alice 4 Sleep System (Respironics Inc., Murrysville, PA, United States). Sleep parameters were evaluated following the 2007 American Academy of Sleep Medicine (AASM) protocol (Berry et al., 2012). Apnea-Hypopnea Index (AHI - the number of apneas or hypopneas recorded during the study per hour of sleep) was derived from level 1 polysomnography (PSG). Cessations of nasal flow lasting 10 or more seconds were defined as apneas, while hypopneas were defines as a decrease of 50% or more in nasal flow and associated with 3% or more oxygen desaturation. The patients with AHI between 5 and 14 were considered to have mild sleep apnea, with AHI between 15 and 29 were considered to have moderate sleep apnea, while the patients with AHI 30 and more were considered to have severe sleep apnea. Oxygen Desaturation Index (ODI), as an index of nocturnal hypoxemia, was derived from the nocturnal pulse oximeter (NPO). The ODI was defined as the number of episodes of oxygen desaturation per hour of sleep, with oxygen desaturation defined as a decrease in blood oxygen saturation (SpO2) to lower than 3% below baseline. The baseline value was determined as the average SpO2 during the first 3 min of recording. Epworth sleepiness scale was used to measure the level of daytime sleepiness. Results from all sleep studies were analyzed by trained personnel.

### Demographic, Laboratory, Respiratory and Cardiovascular Parameters

Demographic data (age, gender, BMI), past medical history (hypertension, diabetes mellitus, hyperlipidemia, coronary heart disease), medications, and data regarding smoking alcohol consumption, and physical activity were collected. Laboratory markers of inflammation and lipid levels were collected, including C-reactive protein, erythrocyte sedimentation rate (ESR), triglycerides, total cholesterol, low-density lipoprotein (LDL), high-density lipoprotein (HDL), non-high-density lipoprotein (non-HDL) and monocyte to HDL ratio. Average and minimum oxygen saturation and percentage of time with oxygen saturation below 90% during the sleep test were registered. The echocardiographic study was performed in all participants on Toshiba TUS-AT Aplio 900/AE machine (Canon Medical Systems Corporation, Otawara, Japan), while 24-h monitoring of arterial blood pressure was performed on MEDILOG AR12PLUS (*Schiller AG*, Baar, Switzerland) and card(X)plore (*Meditech Ltd.*, Budapest, Hungary). The collected data included the following echocardiographic parameters: aortic root diameter, left atrium diameter, end-diastolic and end-systolic left ventricle diameter, interventricular septum thickness, posterior wall thickness, ejection fraction, fractional shortening. Aortic root diameter was measured at the level of Valsalva’s sinuses by M-mode tracings. Mean heart rate during day and night, minimum and maximum heart rate, mean systolic blood pressure, mean diastolic blood pressure, and mean arterial pressure during day and night, as well as mean pulse pressure during day and night, were collected from 24-h blood pressure monitoring. The parameters of arterial stiffness, Augmentation Index (AIx), and Pulse Wave Velocity (PWV), were also calculated. Reference values for laboratory, respiratory, and cardiovascular parameters are provided in Supplementary Table S1.

### Statistical Analysis

Numerical data were presented as mean with 95% confidence interval, or median with minimum and maximum value. Categorical variables were summarized by absolute numbers with percentages. Correlations between numerical variables, AHI and BMI were assessed by the Pearson correlation coefficient. Multivariate linear regression models were used to assess predictors of AHI and BMI. In all analyses, the significance level was set at 0.05. Statistical analysis was performed using IBM SPSS statistical software (SPSS for Windows, release 25.0, *SPSS*, Chicago, IL).

### Ethics

The study was organized according to the principles of the Declaration of Helsinki of 1975, as revised in 2008 and approved by the Ethics Committee of University Clinical Hospital Center “Bezanijska kosa”.

## Results

The study population included 120 patients, with a mean age of 55.7 ± 13.8 years, mostly male (70.0%). Nineteen patients (16.1%) had an AHI of less than five events per hour, 22.9% had an AHI of at least 5 events per hour, but fewer than 15, and 22.0% of patients had an AHI of at least 15 events per hour but fewer than 30. The majority of patients (39.0%) had an AHI of at least 30 events per hour. More than half (56.7%) of the study population were obese (BMI >30) and 28.7% were overweight (BMI between 25 and 30). Twenty-eight percent of the patients were active smokers, 39.4% were consuming alcohol occasionally or often, and 57.3% were physically inactive. Hypertension was present in 57.5% of the study population, 20.0% had diabetes mellitus, 16.7% of the patients had hyperlipidemia, and 11.7% had an history of myocardial infarction. More than half of the patients (56.7%) were on antihypertensive therapy and 16.7% were receiving hypolipemic therapy. Demographic data, comorbidities, use of medications, lipids and inflammation parameters in the study population, as well as their correlation with AHI and BMI are shown in Table 1, 2. Positive correlation was found between both lipid and inflammatory parameters and AHI and BMI (*p* < 0.05 for all).

**TABLE 1:** Demographic data, comorbidities and use of medications in the study population and their correlation with AHI and BMI.

Variable	Total (*n* = 120)	AHI r	BMI r
Age, mean (95% CI), years	55.7 (53.3–58.2)	0.074	0.032
Male gender, n (%)	84 (70.0)	−0.200*	−0.056
BMI>30.0, n (%)	68 (56.7)	0.562**	—
Smoking, yes, n (%)	32 (28.3)	−0.017	−0.148
Alcohol consumption, yes, n (%)	41 (39.4)	−0.110	−0.134
Physically inactive, yes, n (%)	55 (57.3)	−0.167	−0.125
Hypertension, n (%)	69 (57.5)	0.346**	0.320**
Diabetes mellitus, n (%)	24 (20.0)	0.305**	0.296**
Hyperlipidemia, n (%)	20 (16.7)	0.065	0.113
History of myocardial infarction, n (%)	14 (11.7)	0.088	−0.027
Therapy, n (%)			
Antihypertensives	68 (56.7)	0.190*	0.215*
Diuretics	24 (20.0)	0.086	0.219*
Hypolipemic	19 (15.8)	0.016	0.009
Oral antidiabetics	16 (13.3)	0.211*	0.250**
Insulin	7 (5.8)	0.314**	0.258**

BMI, body mass index.

*p < 0.05; **p < 0.01.

**TABLE 2:** Glucose, lipids and inflammation parameters of the study population and their correlation with AHI and BMI.

Variable	Total (*n* = 120)	AHI r	BMI r
Glucose, mean (95% CI), mmol/L	6.3 (5.9–6.8)	0.365**	0.335**
Triglyceride, mean (95% CI), mmol/L	2.0 (1.8–2.2)	0.418**	0.424**
Total cholesterol, mean (95% CI), mmol/L	5.6 (5.4–5.8)	0.019	−0.036
HDL, mean (95% CI), mmol/L	1.2 (1.2–1.3)	−0.334**	−0.482**
LDL, mean (95% CI), mmol/L	3.4 (3.2–3.6)	−0.003	−0.033
Non-HDL, mean (95% CI), mmol/L	4.3 (4.1–4.5)	0.106	0.097
Total cholesterol/HDL-c, mean (95% CI)	4.7 (4.5–4.9)	0.320**	0.445**
LDL-c/HDL-c, mean (95% CI)	2.9 (2.7–3.0)	0.195*	0.327**
CRP, median (25th-75th percentile), mg/L	2.4 (1.3–5.9)	0.241**	0.208*
ESR, median (25th-75th percentile), mm/h	15.5 (8.0–25.0)	0.194*	0.352**
Monocyte/HDL ratio, mean (95% CI)	0.44 (0.40–0.48)	0.318**	0.351**

HDL, high-density lipoprotein; LDL, low-density lipoprotein; CRP, C-reactive protein; ESR, erythrocyte sedimentation rate.

*p < 0.05; **p < 0.01.

Respiratory and cardiovascular parameters of the study population and their correlation with AHI and BMI are shown in Table 3-5. Positive correlation was found between both respiratory and cardiovascular parameters and AHI and BMI (*p* < 0.05 for all).

**TABLE 3:** Respiratory parameters of the study population and their correlation with AHI and BMI.

Variable	Total (*n* = 120)	AHI r	BMI r
% of measurement<90%, mean (95% CI)	17.6 (12.9–22.3)	0.580**	0.553**
Epworth scale, mean (95% CI)	10.7 (9.6–11.9)	0.453**	0.409**
ODI, mean (95% CI)	33.9 (27.5–40.2)	0.957**	0.560**
Average saturation, mean (95% CI), %	90.4 (87.7–93.0)	−0.492**	−0.224*
Min SaO2, mean (95% CI), %	77.5 (75.0–80.0)	−0.708**	−0.594**

ODI, Oxygen desaturation index.

*p < 0.05; **p < 0.01.

**TABLE 4:** 24-h blood pressure monitoring parameters of the study population and their correlation with AHI and BMI.

Variable		Total (*n* = 120)	AHI	BMI
			r	r
SBP, mm/Hg	Total	122.5 (120.3–124.6)	0.137	0.097
	Day	123.3 (121.2–125.5)	0.094	0.046
	Night	116.9 (113.9–119.9)	0.318**	0.367**
DBP, mm/Hg	Total	76.7 (74.7–78.6)	0.017	−0.105
	Day	77.8 (75.8–79.8)	0.015	−0.125
	Night	70.4 (68.1–72.7)	0.038	0.110
MAP, mm/Hg	Total	97.6 (95.7–99.5)	0.084	−0.005
	Day	98.7 (96.7–100.6)	0.059	−0.046
	Night	91.7 (89.2–94.2)	0.197	0.263*
Heart rate, n/min	Total	74.0 (71.8–76.1)	0.204*	0.213*
	Day	76.3 (74.1–78.5)	0.189	0.153
	Night	65.9 (63.3–68.4)	0.385**	0.292**
Pulse pressure, mm/Hg	Total	45.9 (44.4–47.3)	0.171	0.287**
	Day	45.7 (44.2–47.1)	0.114	0.226*
	Night	46.4 (44.3–48.5)	0.415**	0.407**
Pulse pressure, mm/Hg	Total	45.9 (44.4–47.3)	0.171	0.287**
	Day	45.7 (44.2–47.1)	0.114	0.226*
	Night	46.4 (44.3–48.5)	0.415**	0.407**
Aix	Total	20.9 (19.4–22.4)	0.185	0.139
	Day	21.0 (19.6–22.4)	0.184	0.145
	Night	20.9 (18.5–23.4)	0.264*	0.144
PWV	Total	8.3 (7.8–8.7)	0.099	0.068
	Day	8.3 (7.9–8.8)	0.089	0.061
	Night	7.9 (7.3–8.5)	0.113	0.064

SBP, systolic blood pressure; DBP, diastolic blood pressure; MAP, mean arterial pressure; AIx, Augmentation Index; PWV, Pulse Wave Velocity.

*p < 0.05; **p < 0.01.

**TABLE 5:** Echocardiographic parameters of the study population and their correlation with AHI and BMI.

Variable	Total (*n* = 120)	AHI r	BMI r
Aortic root diameter, cm	3.2 (3.2–3.3)	0.366**	0.268**
Left atrium diameter, cm	3.9 (3.8–4.0)	0.294**	0.372**
EDD LV, cm	5.2 (5.1–5.3)	0.292**	0.238*
ESD LV, cm	3.4 (3.2–3.5)	0.309**	0.358**
IVS thickness, cm	1.1 (1.0–1.1)	0.391**	0.455**
PW thickness, cm	1.0 (0.98–1.02)	0.338**	0.426**
RV, cm	2.4 (2.3–2.5)	0.182	0.228*
EF, %	60.6 (59.2–62.0)	−0.354**	−0.270*
FS, %	33.6 (32.0–35.3)	−0.298	−0.201

EDD LV, end-diastolic diameters of left ventricle; ESD LV, end-systolic diameter of left ventricle; IVS, interventricular septum; PW, posterior wall; RV, right ventricle; EF, ejection fraction; FS, fractional shortening.

*p < 0.05; **p < 0.01.

**FIGURE 1:**
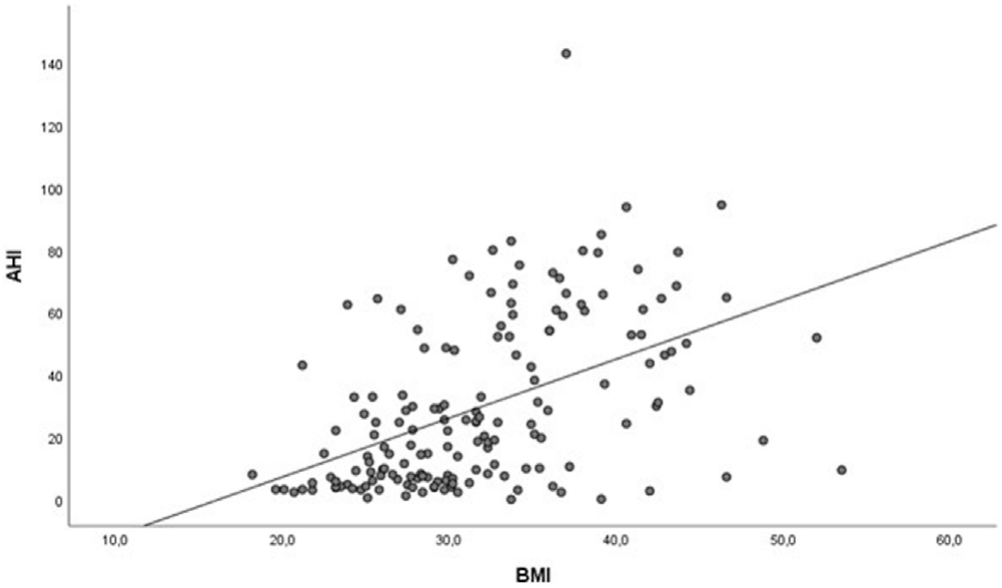
Correlation between AHI and BMI

A strong positive correlation found between AHI and BMI (r = 0.562) is presented in Figure 1.

In a multivariate linear regression model with AHI as a dependant variable, out of demographic data and comorbidities of the study population, hypertension (*p* = 0.004), diabetes mellitus (*p* = 0.038) and gender (*p* = 0.038) were significantly associated with AHI (Table 6). Clinical parameters, such as triglycerides (*p* = 0.002), glucose (*p* = 0.003), and CRP were significant predictors of AHI, while out of respiratory parameters, ODI (*p* < 0.001) and average saturation (*p* < 0.001) were significantly associated with AHI (Table 6). Heart rate at night (*p* = 0.002), pulse pressure at night (*p* = 0.004) and aortic root diameter (*p* < 0.001) were found to be significantly associated with AHI in multivariate linear regression model (Table 6).

**TABLE 6:** Multivariate linear regression model with AHI as a dependent variable.

Variable	B	SE	*p*
Hypertension	14.935	5.138	0.004
Diabetes mellitus	13.205	6.297	0.038
Male gender	−10.670	5.089	0.038
Triglycerides	8.522	2.616	0.002
Glucose	3.004	0.983	0.003
CRP	0.722	0.331	0.032
ODI	0.853	0.025	<0.001
Average saturation	0.374	0.058	<0.001
Heart rate/night	0.892	0.281	0.002
Pulse pressure/night	1.000	0.336	0.004
Aortic root diameter	30.9	7.9	<0.001

CRP, C-reactive protein; ODI, Oxygen Desaturation Index.

In a multivariate linear regression model with BMI as a dependant variable, hypertension (*p* < 0.001) and diabetes mellitus (*p* < 0.001) were significantly associated with BMI (Table 7). Out of clinical parameters, HDL (*p* < 0.001), ESR (*p* = 0.001) and glucose (*p* = 0.016) were significantly associated with BMI. ODI (*p* = 0.029), SaO2 min (*p* < 0.001) and Epworth (*p* = 0.045) were respiratory parameters significantly associated with BMI (Table 7). Heart rate at night (*p* = 0.001) and IVS (*p* = 0.018) were also found to be significantly associated with BMI in multivariate linear regression model (Table 7).

**TABLE 7:** Multivariate linear regression model with BMI as a dependent variable.

Variable	B	SE	*p*
Hypertension	3.369	1.298	<0.001
Diabetes mellitus	3.481	1.604	<0.001
HDL	−8.016	1.657	<0.001
ESR	0.159	0.046	0.001
Glucose	0.536	0.219	0.016
ODI	0.044	0.020	0.029
SaO2 min	−0.236	0.053	<0.001
Epworth	0.182	0.090	0.045
Heart rate/night	27.444	7.679	0.001
IVS thickness	0.178	0.073	0.018

HDL, high-density lipoprotein; ESR, erythrocyte sedimentation rate; ODI, Oxygen Desaturation Index; IVS, interventricular septum.

**FIGURE 2:**
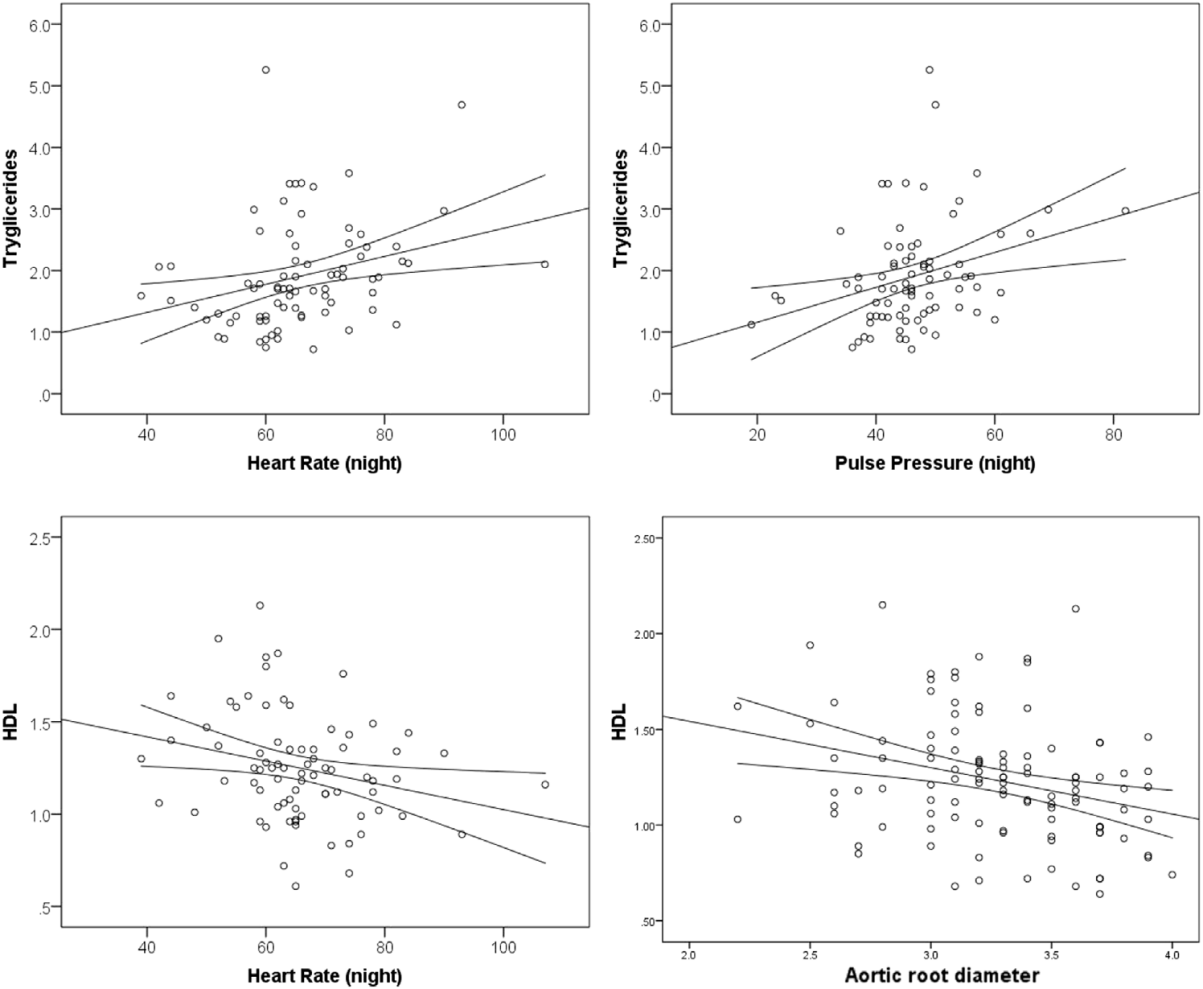
Correlation between tryglicerides, HDL and cardiovascular parameters.

When assessing correlation between lipids and cardiovascular parameters, positive correlation was found between tryglicerides and heart rate during night (r = 0.300; *p* = 0.008), pulse pressure during night (r = 0.307; *p* = 0.006), and IVS thickness (r = 0.231; *p* = 0.024). HDL-c was negatively correlated with heart rate during night (r = -0.255; *p* = 0.024), aortic root diameter (r = -0.296; *p* = 0.002), but also with end-systolic left ventricle diameter (r = -0.270; *p* = 0.008), IVS thickness (r = -0.271; *p* = 0.007), and PW thickness (r = -0.270; *p* = 0.007). IVS was positively correlated with total cholesterol to HDL ratio (r = 0.284; *p* = 0.005) and LDL-c to HDL-c ratio (r = 0.219; *p* = 0.033), while EF was negatively correlated with total cholesterol to HDL ratio (r = -0.274; *p* = 0.012) and LDL-c to HDL-c ratio (r = -0.336; *p* = 0.002). Positive correlation was found between total cholesterol to HDL ratio and PW thickness (r = 0.212; *p* = 0.038) (Figure 2).

**FIGURE 3:**
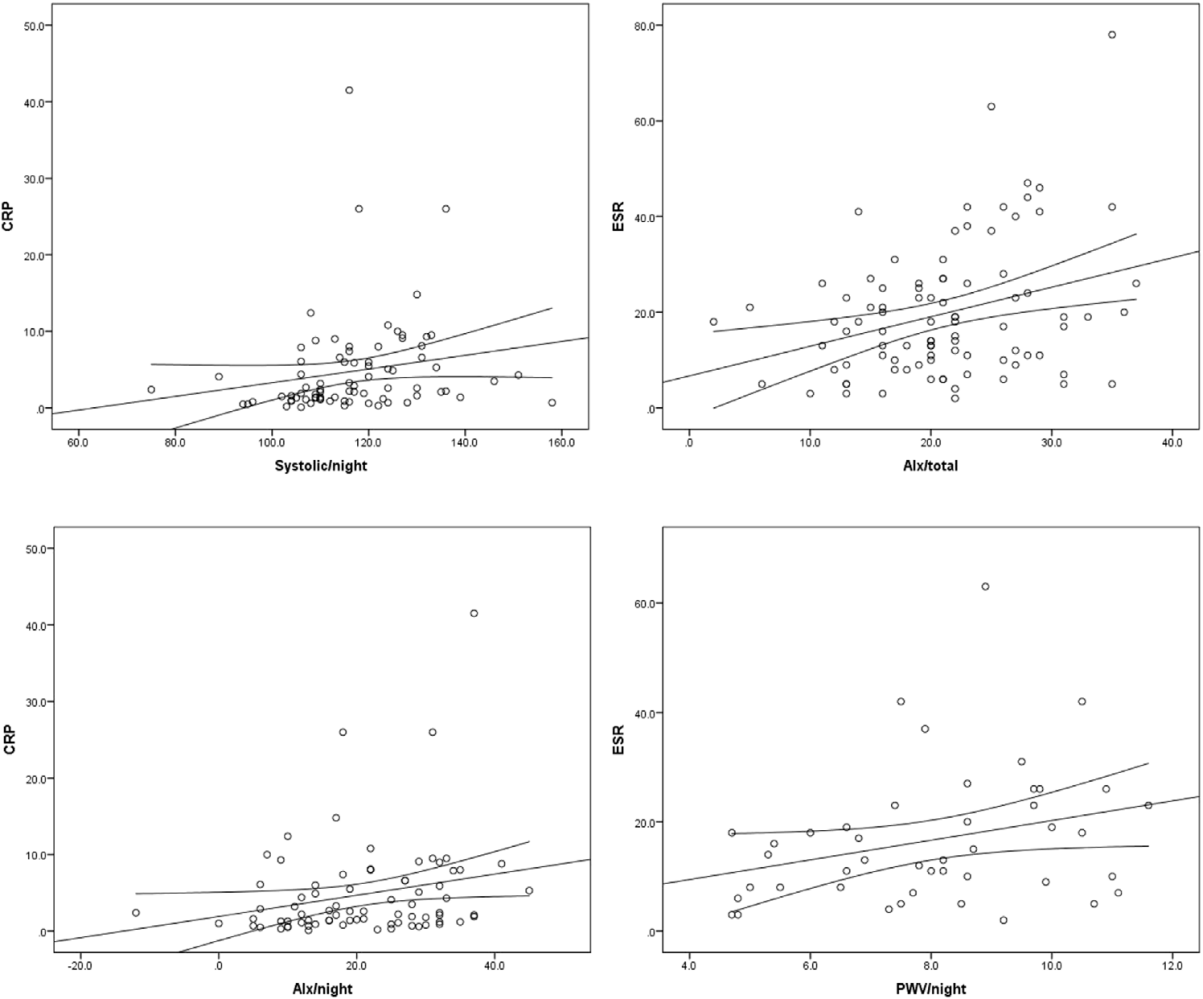
Correlation between CRP, ESR and cardiovascular parameters.

When assessing correlation between inflammatory markers and cardiovascular parameters, positive correlation was found between CRP and mean total systolic blood pressure (r = 0.228; *p* = 0.027), mean systolic blood pressure at night (r = 0.357; *p* = 0.001), mean total heart rate (r = 0.202; *p* = 0.051), mean total heart rate at night (r = 0.253; *p* = 0.024), mean total pulse pressure (r = 0.300; *p* = 0.003), mean total pulse pressure at night (r = 0.437; *p*=<0.001), mean total Augmentation Index (r = 0.217; *p* = 0.036), mean Augmentation Index at day (r = 0.231; *p* = 0.025), mean Augmentation Index at night (r = 0.250; *p* = 0.028), and IVS thickness (r = 0.205; *p* = 0.043). ESR was in positive correlation with mean systolic blood pressure at night (r = 0.267; *p* = 0.018), mean total pulse pressure (r = 0.216; *p* = 0.039), mean pulse pressure at night (r = 0.434; *p* < 0.001), mean total Augmentation Index (r = 0.250; *p* = 0.017), mean Augmentation Index at day (0.274; *p* = 0.009), and mean Pulse Wave Velocity at night (r = 0.312; *p* = 0.039). Positive correlation was also found between monocyte to HDL ratio, AHI (r = 0.236; *p* = 0.002), mean total heart rate (r = 0.219; *p* = 0.035), mean heart rate at night (r = 0.256; *p* = 0.024), and IVS thickness (r = 0.210; *p* = 0.040) (Figure 3).

## Discussion

In the present study on 120 patients with newly diagnosed OSA, we observed a strong correlation between AHI, obesity, lipid abnormalities, inflammatory markers and various cardiovascular parameters. These results are in accordance with previous studies, and provide an insight into potential pathophysiological mechanisms regarding the increased cardiovascular risk in patients with OSA.

OSA is a common condition associated with an increased cardiovascular risk (Archontogeorgis et al., 2018). Intermittent hypoxia in patients with OSA promotes oxidative stress, systemic inflammation, increased lipid production, and endothelium dysfunction, contributing to the development of atherosclerotic cardiovascular disease. It is shown that OSA is an independent risk factor for hypertension, coronary artery disease, type 2 diabetes mellitus, and diabetic kidney disease (Tahrani, 2017; Strausz et al., 2018). Patients with OSA are more susceptible to heart failure, arrhythmias, myocardial infarction, stroke, and sudden death (Roca et al., 2015; Blackwell et al., 2019). Regarding our study population, we observed a significantly higher number of participants with obesity, prior hypertension, coronary artery disease, and diabetes mellitus when compared to the general population (Saeedi et al., 2019; Khan et al., 2020; Mills et al., 2020).

Obesity plays an important role in the pathophysiology of OSA. It is shown that increased body mass index in patients with OSA is strictly related to cardiovascular risk, unlike AHI, as presented in the study by Carratù et al. (2021). Obese patients with OSA have decreased desaturation depth, significantly affecting other respiratory parameters, as presented in our study. We determined that almost every respiratory parameter recorded, including ODI and Epworth scale value, was in correlation with both AHI and BMI. OSA may also worsen the effect of obesity on cardiometabolic risk. *Gasa et al.* showed that OSA in extremely obese patients was associated with major metabolic impairment caused by poorer hypertension, lipid, and glucose control (Gasa et al., 2011). We presented a strong correlation between glucose levels, lipid abnormalities, inflammatory markers, and both AHI and BMI, even in multivariate logistic regression model, revealing that patients with severe OSA and increased BMI may have more pronounced low-grade systemic inflammation and metabolic syndrome, which can lead to increased cardiovascular risk in these patients. The presence of hypertension and diabetes mellitus was also significantly associated with AHI and BMI. However, it is important to note that this correlation was significant even in patients on antihypertensive and antidiabetic therapy.

Recurrent hypoxia, continuous stimulation of the renin-angiotensin-aldosterone system, and sympathetic nervous system activation promote hypertension development in patients with OSA. Hypertension in patients with OSA is characterized by nocturnal hypertension, abnormal blood pressure variability, and relatively poor regulation with standard antihypertensive therapy (Pio-Abreu et al., 2021). These abnormal patterns of blood pressure variation are associated with advanced target organ damage and poor cardiovascular prognosis. In our study, 57.5% of patients had prior hypertension, while the majority of blood pressure parameters registered at night (mean systolic blood pressure and mean pulse pressure) were associated with AHI, as well as with several lipid and inflammatory markers, including triglycerides, CRP, and ESR. *Dudenbostel et al.* reported that 90% of male patients and 77% of female patients with resistant hypertension had obstructive sleep apnea, while nocturnal hypertension is significantly higher in patients with OSA and is associated with the risk of total cardiovascular disease, cardiovascular death, heart failure, stroke and myocardial infarction (Dudenbostel and Calhoun, 2012). A 20 mmHg increase in night-time systolic blood pressure was significantly associated with a 36% increase in cardiovascular event risk and a 23% increase in mortality (Al Ghorani et al., 2021). Effective control of co-morbidities, optimal antihypertensive drug treatment, continuous positive airway pressure therapy, and other therapeutic modalities are substantial in improving hypertension control and reducing cardiovascular risk. The potential benefit of renal sympathetic denervation, as a novel therapeutic modality, is important in these patients, as this procedure can provide better long-term blood pressure regulation and improve respiratory parameters (Warchol-Celinska et al., 2018). It is shown that inhibition of sympathetic nerve activity after renal denervation procedure is associated with a reduction of monocyte activation and other inflammatory markers in patients with resistant hypertension (Zaldivia et al., 2017). In the study by *Jung et al.*, patients with obstructive sleep apnea had significantly higher values of CRP, which was marked as an independent risk predictor for cardiovascular morbidity (Jung et al., 2021). Several other inflammatory markers were identified as important in patients with obstructive sleep apnea and hypertension, including plasma monocyte chemoattractant protein-1, interleukin-1β, tumor necrosis factor-α, and interleukin-12 (Baessler et al., 2013).

Up to 50% of patients with OSA have nocturnal arrhythmias, mostly atrial fibrillation and ventricular arrhythmias (Hersi, 2010). Arrhythmias are emphasized by intermittent hypoxia and sympathetic nervous system activation and are associated with an increased risk of sudden cardiac death during sleep (Ludka et al., 2011). Although we did not perform 24h ECG Holter monitoring, it is notable that mean heart rate values, especially at night, were higher in patients with severe OSA and in correlation with almost every lipid and inflammatory parameter. This conclusion can be explained mainly as a result of increased nocturnal intermittent hypoxia initiating oxidative stress and sympathetic nervous system over-activation, causing low-grade systemic inflammation and increased cholesterol production with decreased clearance. Even monocyte to HDL ratio, a novel marker which is prooven be an important predictor of increased cardiovascular risk, had a significant correlation with AHI, mean total heart rate, and mean heart rate at night. Several studies have suggested that heart rate has a negative effect on cardiovascular morbidity and mortality and is associated with cardiac remodeling, increased vascular stiffness, dyslipidemia, obesity, insulin resistance, and atherosclerosis (Tadic et al., 2018).

Obstructive sleep apnea is significantly associated with cardiac remodeling, leading to atrial dilatation, left ventricular hypertrophy, enlargement, mass increase, diastolic dysfunction, and reduction of systolic function (Yu et al., 2020). In our study, AHI was associated with several echocardiographic parameters including aortic root diameter, left atrium diameter, end-diastolic and end-systolic left ventricle diameter, interventricular septum thickness, posterior wall thickness, and ejection fraction. The study by Aslan et al. showed that certain left ventricular functional alterations, such as left ventricular hypertrophy, left ventricular diastolic dysfunction, and left atrial dilatation, occur even before the development of hypertension in patients with OSA (Aslan et al., 2013). As reported by *Maripov et al.*, cardiac remodeling also involves the right ventricle in terms of right ventricular dilatation, increased wall thickness, and altered right ventricular function (Maripov et al., 2017). Our study suggested a strong negative correlation between HDL cholesterol and aortic root diameter, end-systolic left ventricle diameter, IVS thickness, and PW thickness, while EF was negatively correlated with total cholesterol to HDL ratio. It seems that echocardiographic parameters in patients with OSA are strongly associated with lipid profile than with inflammatory markers, as only IVS thickness was associated with CRP and monocyte to HDL ratio. In addition to their established role in atherosclerosis, certain studies suggested a significant relationship of lipid abnormalities with adverse changes in cardiac structure and function (Aung et al., 2020). A low value of HDL cholesterol is associated with left ventricle remodeling and diastolic dysfunction, especially in patients with hypertension, which is significant as the majority of the patients with OSA have it (Horio et al., 2003). Regarding the potential benefits of lipid-lowering therapy, it is shown that high-dose statin therapy can improve blood lipid metabolism, reduce the inflammatory response, and prevent and treat ventricular remodeling and myocardial fibrosis (Luo et al., 2020). These positive effects can significantly reduce cardiovascular risk and improve further prognosis.

Arterial stiffness is a strong independent predictor of adverse cardiovascular events and mortality (Mitchell et al., 2010). Various studies demonstrated the association between parameters of arterial stiffness, Augmentation Index, and Pulse Wave Velocity, with the severity of obstructive sleep apnea (Joyeux-Faure et al., 2018; Theorell-Haglöw et al., 2019). In our study, the mean total Augmentation Index was significantly associated with CRP and ESR, while mean Pulse Wave Velocity at night was associated only with ESR. However, only the mean Augmentation Index at night was significantly associated with AHI, as well as with ESR. Certain studies showed that apnea parameters in patients with OSA have little independent influence on arterial stiffness and are mainly driven by standard risk factors, including age, body mass index, systolic blood pressure, and diabetes mellitus (Tamisier et al., 2016). It is shown that inflammation plays an important role in the development of arterial stiffness, while inflammatory markers could be significant in the assessment of cardiovascular risk. Various inflammatory markers revealed a significant correlation with the severity of arterial stiffness, including white blood cell count, neutrophil to lymphocyte ratio, adhesion molecules, fibrinogen, C-reactive protein, cytokines, microRNAs, and cyclooxygenase-2 (Mozos et al., 2017). Low-grade systemic inflammation in patients with OSA potentiates vascular inflammation, endothelial dysfunction, and consequent atherosclerosis. Understanding the role of inflammation in the pathogenesis of arterial stiffness can provide significant information to develop useful therapeutic modalities and potentially reduce cardiovascular risk in these patients.

Aortic root diameter was significantly associated with AHI in the multivariate linear regression model. A similar conclusion was established in several studies investigating the relation between aortic root diameter and the severity of OSA (Lee et al., 2010; Baguet et al., 2011). The study by *Delsart et al.* demonstrated that the relationship between aortic root diameter and the severity of OSA persisted even in patients on CPAP therapy (Delsart et al., 2019). It is important to note that an increased aortic root diameter represents a risk factor for left ventricular hypertrophy, left ventricular dysfunction, and renal dysfunction (Wang et al., 2022). An increased aortic root diameter can lead to progressive aortic root dilation, which can be a significant risk factor for major adverse cardiovascular events and mortality (Gaisl et al., 2015). The pathophysiology of aortic root dilation in patients with OSA is still unclear. However, the underlying mechanisms could involve intermittent hypoxia and reoxygenation, increased sympathetic activity, and increased wall stress against intrathoracic organs, especially in hypertensive patients with OSA. The study by *Alegret et al.* demonstrated that cholesterol is associated with increased aorta diameter and dilation (Alegret et al., 2015). *Chen et al.* concluded that lipid profile had no relation to any aortic segments diameter, except HDL cholesterol that had a significantly positive effect on the diameter of ascending aorta (Chen et al., 2022). In our study, aortic root diameter was in negative correlation only with HDL, meaning that patients with lower HDL values had larger aortic root diameter. We observed no significant correlation with other lipid values or inflammatory parameters. This is important as it is still undetermined whether the dyslipidemia is in relationship with aortic root dilation by consequently emphasizing the atherosclerotic process. Certain studies demonstrated that increased aortic root diameter in patients with OSA is weakly dependent on atherosclerosis (Agmon et al., 2003).

The present study has several advantages and limitations. It represents not only strong evidence of the association between various cardiovascular parameters and OSA severity but also the association with lipid abnormalities and inflammation, potentially revealing the main pathophysiological mechanisms responsible for increased cardiovascular risk. The novelty of this paperwork lies in the fact that according to common cardiovascular parameters that we observe in everyday clinical practice, we can stratify OSA patients with an increased risk of developing cardiovascular complications. Newly diagnosed patients with the severe form of obstructive sleep apnea, lipid abnormalities, and higher levels of inflammatory parameters, associated with other significant risk factors, should go through an extensive cardiovascular screening to prevent future adverse cardiovascular events. It is also important to note that by including only patients with newly diagnosed obstructive sleep apnea we prevented the possible positive effects of CPAP therapy on the improvement of cardiovascular parameters and reduction of cardiovascular risk. The main limitations of the study are the lack of 24-h ECG Holter monitoring data, the scarce number of evaluated inflammatory parameters, and the absence of follow-up regarding future adverse cardiovascular events and outcomes.

## Conclusion

In the present study on 120 patients with newly diagnosed OSA, we observed a significant correlation between OSA severity, obesity, lipid abnormalities, inflammatory markers, and various cardiovascular parameters. These results imply the inevitable role of dyslipidemia and systemic inflammation with the occurrence of atherosclerotic cardiovascular disease in patients with OSA, leading to an increased susceptibility for coronary artery disease, myocardial infarction, arrhythmias, stroke, and sudden death. Future studies should focus on the identification of credible risk predictors and the development of novel therapeutic modalities to significantly reduce the chance of future major adverse cardiovascular events.

## Data Availability

The raw data supporting the conclusion of this article will be made available by the authors, without undue reservation.

## Abbreviations

AHI, apnea-hypopnea Index; AIx, augmentation index; BMI, body mass index; CPAP, continuous positive airway pressure; CRP, C-reactive protein; DBP, diastolic blood pressure; EDD LV, end-diastolic diameter of left ventricle; EF, ejection fraction; ESD LV, end-systolic diameter of left ventricle; ESR, erythrocyte sedimentation rate; FS, fractional shortening; HR, heart rate; IVS, interventricular septum; MAP, mean arterial pressure; OSA, obstructive sleep apnea; PSG, polysomnography; PW, posterior wall; PWV, pulse wave velocity; RV, right ventricle; SBP, systolic blood pressure.
