# Supplementary material for: Obstructive Sleep Apnea and Cardiovascular Risk: The Role of Dyslipidemia, Inflammation, and Obesity
Source: Front Pharmacol. 2022 Jun 15;13:898072. doi: 10.3389/fphar.2022.898072 (PMC9240428; doi:10.3389/fphar.2022.898072)
Supplement: Supplementary file 1 [file Table1.DOCX]

**Supplemental Table 1.**

Reference values and scores of evaluated indices, laboratory, and cardiovascular parameters

| **Body mass index (BMI, kg/m^2^)** | |
| --- | --- |
| Underweight | <18.5 |
| Normal range | 18.5-24.9 |
| Overweight | 25-29.9 |
| Obese | ≥30.0 |
| Class I | 30.0-34.9 |
| Class II | 35.0-39.9 |
| Class III | ≥40.0 |
| **Oxygen Desaturation Index (ODI) scoring** | |
| <5 | Normal (no sleep apnea) |
| ≥5-14 | Mild sleep apnea |
| 15-29 | Moderate sleep apnea |
| ≥30 | Severe sleep apnea |
| **Apnea-Hypopnea Index (AHI) scoring** | |
| <5 | Normal (no sleep apnea) |
| ≥5-14 | Mild sleep apnea |
| 15-29 | Moderate sleep apnea |
| ≥30 | Severe sleep apnea |
| **Epworth Sleepiness Scale** | |
| 0-10 | Normal range |
| 11-14 | Mild sleepiness |
| 15-17 | Moderate sleepiness |
| 18-24 | Severe sleepiness |
| **Reference values of laboratory parameters** | |
| **Biochemical parameters** | |
| C-reactive protein | up to 5 mg/L |
| Triglycerides | up to 1.7 mmol/L |
| Total cholesterol | up to 5.2 mmol/L |
| Low-density lipoproteins (LDL) | up to 3.4 mmol/L |
| High-density lipoproteins (HDL) | up to 1.6 mmol/L |
| Fibrinogen | 2.1-4.0 g/L |
| **Complete blood count** | |
| Leukocytes | 4.0-10.0x10^9^/L |
| Erythrocytes | 4.5-6.5/10^12^/L in men, 3.8-5.8/10^12^/L in women |
| Hemoglobin | 130-170g/L in men, 115-160g/L in women |
| Hematocrit | 0.40-0.54L/L in men, 0.37-0.47L/L in women |
| Thrombocytes | 150.0-450.0x10^9^/L |
| Neutrophils | 2.0-7.5x10^9^/L |
| Lymphocytes | 1.0-4.0x10^9^/L |
| Monocytes | 0.2-1.0x10^9^/L |
| Eosinophils | up to 0.5x10^9^/L |
| Basophils | up to 0.2x10^9^/L |
| Erythrocyte sedimentation rate (ESR) | up to 12mm/h in men, up to 19mm/h in women |
| **Respiratory parameters** | |
| Oxygen saturation (SpO2) | >95% |
| Forced Vital Capacity (FVC) | 80-120% |
| Forced Expiratory Volume in 1 second (FEV1) | 80-120% |
| Forced Expiratory Volume to Vital Capacity Ratio (FEV1/FVC) | ≥70% |
| pCO2 | 4.7-6.0 kPa |
| pO2 | 10.3-13.3 kPa |
